# Hydration Repulsion between Carbohydrate Surfaces Mediated by Temperature and Specific Ions

**DOI:** 10.1038/srep28553

**Published:** 2016-06-23

**Authors:** Hsieh Chen, Jason R. Cox, Hooisweng Ow, Rena Shi, Athanassios Z. Panagiotopoulos

**Affiliations:** 1 Aramco Services Company: Aramco Research Center – Boston, Cambridge, MA 02139, USA; 2 Department of Chemical and Biological Engineering, Princeton University, Princeton, NJ 08544, USA.

## Abstract

Stabilizing colloids or nanoparticles in solution involves a fine balance between surface charges, steric repulsion of coating molecules, and hydration forces against van der Waals attractions. At high temperature and electrolyte concentrations, the colloidal stability of suspensions usually decreases rapidly. Here, we report a new experimental and simulation discovery that the polysaccharide (dextran) coated nanoparticles show ion-specific colloidal stability at high temperature, where we observed enhanced colloidal stability of nanoparticles in CaCl_2_ solution but rapid nanoparticle-nanoparticle aggregation in MgCl_2_ solution. The microscopic mechanism was unveiled in atomistic simulations. The presence of surface bound Ca^2+^ ions increases the carbohydrate hydration and induces strongly polarized repulsive water structures beyond at least three hydration shells which is farther-reaching than previously assumed. We believe leveraging the binding of strongly hydrated ions to macromolecular surfaces represents a new paradigm in achieving absolute hydration and colloidal stability for a variety of materials, particularly under extreme conditions.

Hydration repulsion is the universal force that acts between well-solvated surfaces in water and balances other surface attractions (e.g. van der Waals interactions) in the nanometer range^1^. This force is ubiquitous in biology and solution chemistry such as polymer and protein aggregation, protein folding, enzyme activity, and lubrication^2^,^3^,^4^. Experimentally, the hydration force was first measured from stacked phospholipid multi-bilayers in terms of pressure-distance curves by the osmotic stress technique and X-ray diffraction^5^,^6^,^7^, and later from two individual bilayers by the surface force apparatus^8^. For different systems (lipid bilayers, proteins, DNA double helices, and polysaccharides)^6^,^7^,^9^,^10^,^11^, the measured pressure-distance curves show surprisingly similar exponential decay with a characteristic length scale of ~0.2 to 0.4 nm^12^,^13^ that highlight the universality of the hydration force. Temperature and specific ions may dramatically change the hydration repulsion. At elevated temperature, it was observed that the hydration repulsion decreases, most likely due to temperature-induced dehydration on the surfaces^9^,^14^,^15^. How the specific ions affect hydration repulsion, however, is less clear. It is important to point out that at high salt concentrations the colloidal stability of suspensions usually decreases due to the screening of any electrostatic repulsion; however, this mechanism is non-specific for ions and should be distinguished from the hydration forces. Macroscopically, the decreased hydration repulsion also results in the decreased colloidal stability (and subsequent precipitation) of suspensions. Traditionally, ions have been ordered in the so-called Hofmeister series according to their ability to precipitate (salt out) proteins^16^,^17^,^18^. The emerging view, however, is that ion-specific effects are actually a complex phenomenon involving local interactions between ions and patches or segments at protein surfaces^19^,^20^,^21^. Prior laboratory experiments and molecular simulations have suggested that the surface binding ions may in fact increase solubility (salt in), while non-binding ions indefinitely decrease solubility (salt out) of proteins^22^,^23^,^24^,^25^. Nevertheless, the microscopic mechanism is still largely missing. Specifically, how do the surface bound ions contribute to the extra repulsion? Do the surface bound ions add extra electrostatic repulsion, increase surface hydration, or both? How many hydration shells on macromolecular surfaces would the bound ions perturb, if at all? To this end, we try to answer these fundamental questions in the present study by means of molecular simulations.

Recent elegant work by Cremer, among others, has elucidated the effect of ion binding to macromolecular structures such as poly(*N*-isopropylacrylamide) (PNIPAM) and elastin-like polypeptides (ELPs) in the context of polymer-polymer and polymer-solvent interactions^22^,^23^,^26^. Experiments on the poly(*N*-isopropylacrylamide) and elastin-like polypeptides revealed that weakly hydrated (“chaotropic”) anions such as SCN^−^ and I^−^ tend to bind to the polymers and increase the lower critical solution temperature (LCST) (salt in), while strongly hydrated (“kosmotropic”) anions such as SO_4_^2−^ do not bind to the polymers and decrease the LCST (salt out). The propensity for different anions to increase or decrease the LCST (salt in or salt out) of these thermosensitive polymers generally follows the Hofmeister series^22^,^23^,^26^. Herein, we build upon this previous work by examining the synergistic effect of temperature and specific ions on the colloidal stability of carbohydrate coated magnetic nanoparticles. Namely, we show that polymer-polymer and polymer-solvent interactions can be finely tuned by specific ion effects and that these interactions, in turn, can modulate the aggregation rate of nanoparticles. More surprisingly; however, we observe specific *cation* effects whereby the colloidal stability of dextran coated magnetic nanoparticles is much greater in the presence of Ca^2+^ ions compared to Mg^2+^ ions – a surprising result indeed given the proximity of these two electrolytes in the Hofmeister series.

Despite considerable progress in experimental measurements, the interpretation of the pressure-distance curves between hydrated surfaces in terms of physically distinct force components involves serious complications since the experimental measurements provide only the total magnitude of the force. Such difficulties impart great importance to computer simulations that allow direct evaluation of separate force components through the respective ensemble averages. However, due to computational limitations, the quantitative comparison between experiments and simulations has only been made possible recently^27^,^28^,^29^,^30^. Furthermore, the simulation studies on hydration forces have mainly focused on phospholipid membranes^14^,^29^,^30^,^31^,^32^, and no ion-specific effects have been considered. The carbohydrates studied here, in fact, provide a unique opportunity for exploring the microscopic mechanism for ion-specific effects on hydration repulsion through simulation since carbohydrate-specific ion binding constants have been well-established and realistic simulation force fields may be developed^33^,^34^. In this work, we performed extensive molecular dynamics simulations for carbohydrate surfaces with decreasing distances, capturing detailed hydration repulsion processes (pressure-distance curves) with conditions that resemble our experiments on dextran-coated nanoparticles. To the best of our knowledge, this is the first molecular simulation study which explores how the specific ion binding can affect the pressure-distance curves. We believe the microscopic mechanism derived from simulations combined with macroscopic experiments presented here provide an improved insight into the temperature and ion-specific effect on hydration of carbohydrates, which possesses many technologically as well as biologically relevant implications.

## Results

### Dynamic Light Scattering Measurements for the Hydrodynamic Diameter of Dextran-Coated Nanoparticles in Different Electrolyte Solutions at Different Temperatures

**Figure 1:**
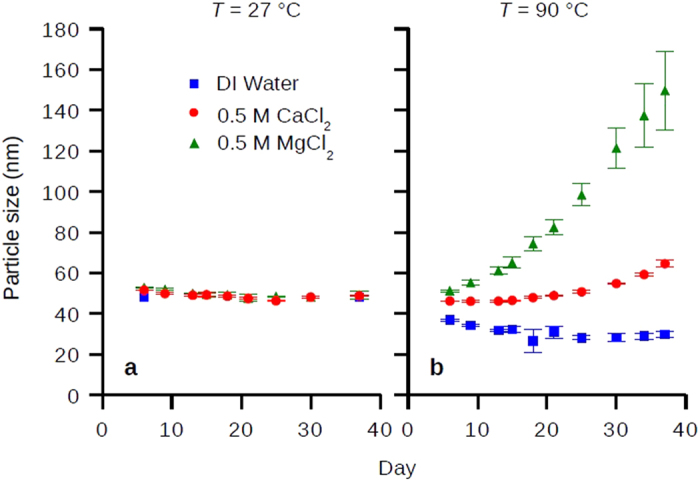
Colloidal stability and swelling behavior of polysaccharide (dextran) coated nanoparticles. Dynamic light scattering data for the hydrodynamic diameters of dextran coated nanoparticles in deionized water, 0.5 M MgCl_2_, and 0.5 M CaCl_2_ over a 40 day period at (**a**) *T* = 27 °C and (**b**) *T* = 90 °C.

The salient effects of specific ions and temperature on the long term colloidal stability of polysaccharide (dextran) coated superparamagnetic nanoparticles (Dex-SPIONs) are illustrated in Fig. 1. In these experiments, the colloidal stability of nanoparticles in 0.5 M MgCl_2_, 0.5 M CaCl_2_ and deionized water was followed via dynamic light scattering (DLS) over a 40 day period at room temperature and at *T* = 90 °C. At *T* = 27 °C, the nanoparticle hydrodynamic diameters in electrolyte solutions and deionized water are all similar (~50 nm) and do not change as a function of time. At *T* = 90 °C, the DLS results stand in stark contrast to the *T* = 27 °C case. In deionized water, the nanoparticle hydrodynamic diameters decrease quickly by ~40% over the first 14 days, indicating that the dextran chains are collapsing onto the surface of the iron oxide cores (or one another) due to polymer dehydration. The nanoparticles in 0.5 M MgCl_2_ solution exhibit a fast growth in hydrodynamic diameter after only a week at 90 °C – a behavior that is indicative of nanoparticle aggregation, presumably due to fast dehydration followed by subsequent nanoparticle-nanoparticle aggregation. Conversely, the nanoparticles in 0.5 M CaCl_2_ solution behave very similarly to the *T* = 27 °C case, suggesting that the dextran units remain well hydrated at elevated temperature thereby preventing nanoparticle aggregation.

### Molecular Dynamics Simulations of the Hydration Repulsion between Carbohydrate Surfaces Mediated by Temperature and Specific Ions

**Figure 2:**
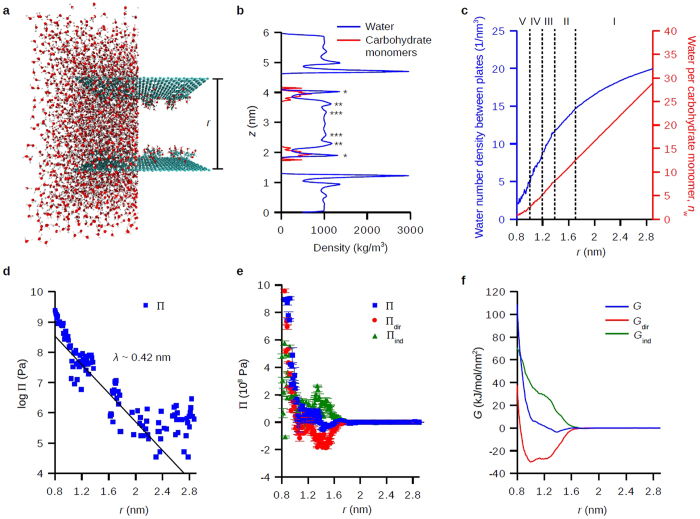
Hydration pressure between carbohydrate surfaces calculated from molecular dynamics simulations. (**a**) Snapshot of simulation setup. Note half of the water molecules in the simulation box are not shown for clarity. (**b**) Density distribution of carbohydrate monomers and waters along surface normal (*z* axis) within the column of *x*-*y* dimensions of the surfaces. The single (*), double (**), and triple stars (***) denote the inner, first, and second hydration layers on carbohydrates. (**c**) Water per carbohydrate monomer, *n*_w_, and water number density between surfaces as a function of inter-surface distance *r*. I–V represent different water depolarization, dehydration, or direct interaction regimes (see Text). (**d**) Net pressure Π acting on carbohydrate surfaces as a function of *r* calculated from simulations plotted in a semi-logarithmic scale. The exponential decay length λ is fitted for Π(*r*) from 1 < *r* < 2 nm. (**e**) Total, direct, and indirect pressure (Π, Π_dir_, and Π_ind_) calculated from simulations. The errors were analyzed from the standard deviation of block averages of simulation time periods 40 to 60, 60 to 80, and 80 to 100 ns. (**f**) Total, direct, and indirect free energy (*G*, *G*_dir_, and *G*_ind_) calculated by integrating the pressures in (**e)** along *r*.

Figure 2a shows a simulation snapshot of carbohydrate monomers (α-D-allopyranose) tethered to surfaces separated by distance *r*, hydrated by simple point charge-extended (SPC/E) water molecules. Each surface has a size of 4 × 4 nm^2^ and 16 tethered carbohydrate monomers, with the area per carbohydrate monomer equal to 1 nm^2^. The initial water box size is 6 × 6 × 6 nm^3^. We used the GROMACS simulation package and a CHARMM based force field for simulating the carbohydrates and surfaces (see Methods). Figure 2b shows the density distribution of water and carbohydrate monomers along the surface normal (*z* axis) within the column of *x*-*y* dimensions of the surfaces, where water structures were clearly seen on the hydrated carbohydrate monomers. The first and second hydration layers on carbohydrates are denoted with double (**) and triple stars (***), while the single star (*) denotes the inner hydration layer between the carbohydrates and their tethering surfaces. When decreasing *r*, the number of waters between surfaces decreases, as shown in Fig. 2c where we calculated the number of waters per carbohydrate monomer *n*_w_ which decreases from ~30 to less than 1 for *r* from 2.9 to 0.8 nm showing strong dehydration at small distances.

To gain a detailed hydration repulsion picture with the resolution of individual water layers, we calculated the hydration pressure Π as a function of *r* (Fig. 2d,e) by performing 211 independent 100 ns simulations with fixed *r* from 2.9 nm to 0.8 nm with 0.01 nm increments. The hydration pressure Π, which is readily measured in experiments^5^,^6^,^7^,^8^, was calculated from the total forces exerted on the position-restrained surfaces divided by their areas, equivalent to the pressure difference between the confined solutions and bulk water. Figure 2d shows Π(*r*) in a semilogarithmic plot in which an exponential decay with decay length λ ~ 0.42 nm is seen for 1 < *r* < 2 nm, and the max pressure within this range is of the order of 10^8^ Pa. We point out that our pressure-distance curve (Fig. 2d) agrees quantitatively with prior experiments in terms of the absolute pressure scale, the exponential decay length, and the shape of the Π(*r*) curve, validating the accuracy of our simulations^12^,^13^. To distinguish the contributions from water and surface molecules, we divided the total pressure into direct and indirect contributions^29^,^30^,^32^. In Fig. 2e we show the direct pressure Π_dir_ between carbohydrate surfaces, calculated using a similar simulation procedure as for Fig. 2d, but in vacuum. As seen, the direct carbohydrate-carbohydrate interaction is attractive except at short distances, and the net repulsion seen in Fig. 2d is from the indirect solution-mediated pressure Π_ind_ = Π − Π_dir_. Notice that Π_dir_ becomes repulsive for *r* < 1 nm because of the strong overlap of the carbohydrates at this small distance. The rapid increase of Π_dir_ for *r* < 1 nm explains why there exists another scaling for the pressure-distance curve for *r* < 1 nm (Fig. 2d). Finally, for thermodynamic analysis we calculated the interaction free energy *G* by integrating the hydration pressure along *r*


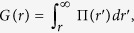


and setting the max distance *r* = 2.9 nm as the reference state. Figure 2f shows the free energy curves for total, direct, and indirect interactions.

**Figure 3:**
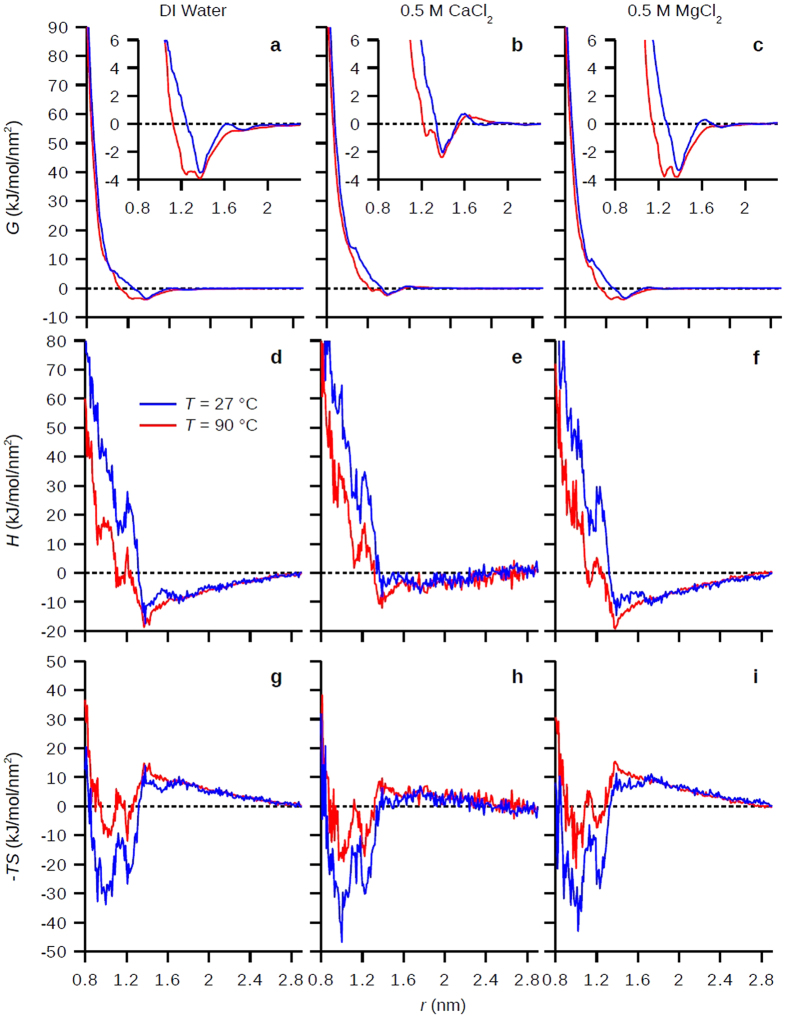
Free energies and enthalpy-entropy contributions for hydration repulsion at different temperatures in different solutions. (**a–c**) Free energies, *G*, (**d–f**) enthalpic contributions, *H*, and (**g–i**) entropic contributions, *-TS*, for the hydration repulsion between carbohydrate surfaces in DI water (**a,d,g**), 0.5 M CaCl_2_ (**b,e,h**), and 0.5 M MgCl_2_ (**c,f,i**) at *T* = 27 or 90 °C.

Figure 3 shows the free energy (*G*), enthalpy (*H*), and entropic contribution (*-TS*) as a function of distance *r* at room temperature (27 °C) and at high temperature (90 °C) in DI water, 0.5 M CaCl_2_, and 0.5 M MgCl_2_. For the electrolyte solutions, the ion-carbohydrate interactions were reparametrized to fit their binding constants (Methods). Enthalpies were approximated by system internal energies because the work of expansion due to variations in total system volume is negligible, and entropic terms were calculated from *-TS* = *G–H*. Along *r* we can identify five regimes: (I) water depolarization at *r* > 1.7 nm, (II) dehydration for the second water layer and carbohydrate-carbohydrate attraction at 1.7 > *r* > 1.4 nm, (III) dehydration for the first water layer at 1.4 > *r* > 1.2 nm, (IV) dehydration for the inner water layer at 1.2 > *r* > 1 nm, and (V) direct repulsion at *r* <1 nm. (For II–IV, see Fig. 2b for different water layers as well as Fig. 2c for the different water number density decaying slopes indicating different dehydration processes; for V, see Π_dir_ in Fig. 2e). The dehydration regimes (II, III, and IV, 1.7 > *r* > 1 nm) are characterized by large increase of *H* (Fig. 3d–f) and large decrease of *-TS* (Fig. 3g–i) when decreasing *r* except in regime II where direct carbohydrate-carbohydrate attractions compensate the enthalpy increase. In terms of temperature effects for dehydration, at *T* = 90 °C we see lower increase of *H* and lower decrease of *-TS*. These compensating energies result in rich free energy curves as shown in Fig. 3a–c. For all cases, *G* decreases about 3~4 kJ/mol/nm^2^ in regime II when decreasing *r* because direct carbohydrate interaction (*G*_dir_ in Fig. 2f) is stronger than the dehydration of second water layer (triple stars in Fig. 2b). The interesting result here is that in regime III we see at *T* = 90 °C the dehydration energy for the first water layer (double stars in Fig. 2b) decreased significantly compared to *T* = 27 °C (Fig. 3a–c insets), shifting the equilibrium distances for the two surfaces ~0.2 nm shorter (except in CaCl_2_ solution, discussed later), which agree well with previous experimental and simulation studies on the temperature-induced dehydration^9^,^14^,^15^. Analyzing the pair interaction potentials between carbohydrates and water molecules shows direct evidence that the carbohydrates are less ‘hydrophilic’ and have lower hydration repulsion at 90 °C (Supplementary Discussion). In regime IV and V, we see that the dehydration of the inner water layer (single star in Fig. 2b) and the direct repulsion increase *G* greatly and the two surfaces are highly repulsive at the short distances.

**Figure 4:**
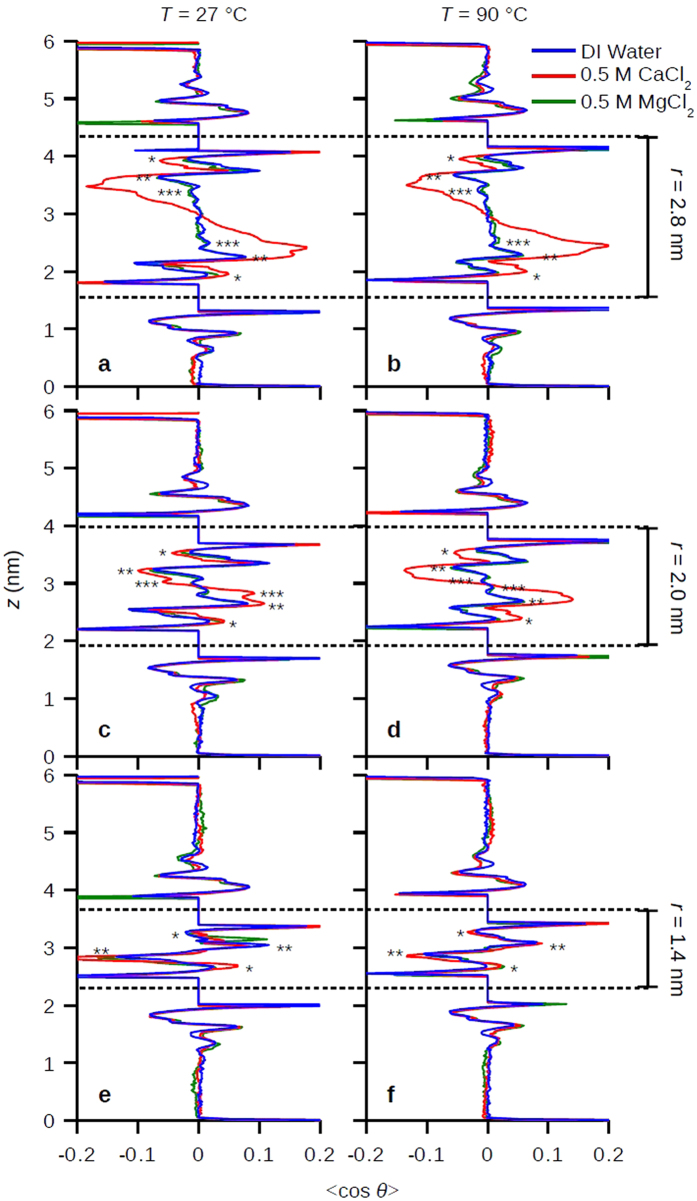
Water polarization profile between carbohydrate surfaces. Water polarization profile for surface distances (**a,b**), *r* = 2.8, (**c,d**) *r* = 2.0, and (**e,f**), *r* = 1.4 nm at *T* = 27 °C (**a**,**c**,**e**) or *T* = 90 °C (**b**,**d**,**f**). The single (*), double (**), and triple stars (***) denote the inner, first, and second hydration layers on carbohydrates which coincide with those in Fig. 2b.

The water depolarization regime I is characterized by the gradual decrease of *H* and increase of *-TS* (Fig. 3d–i, *r* > 1.7 nm) when decreasing *r* because of the cancelation of approaching water polarization profiles from two opposing hydrophilic surfaces^29^,^35^. Figure 4 shows the average water dipole angle projected on *z* axis, <cos *θ* >, along *z* at three different *r*, which all the water profiles are depolarized (<cos *θ*> = 0) in center due to symmetry. For *r* = 2.8 or 2.0 nm which are in the depolarization regime (Fig. 4a–d), in CaCl_2_ solution (where Ca^2+^ ions complex with the carbohydrates) the water polarization profile shows extended and strongly polarized structure beyond at least three hydration shells (~0.75 nm, the max distance from one carbohydrate surface to the midpoint between surfaces in this study), which is farer reaching than previous thought to occur^19^. Conversely, in DI water and MgCl_2_ solution (where there are no ion complexes) the water polarization profiles show weakly perturbed oscillations which coincide with the hydration layers (c.f. Fig. 2b). For *r* = 1.4 nm in the dehydration regime (Fig. 4e,f), the polarization profiles are similar in all solutions where the peaks for the hydration layers from both surfaces intermixed with each other. The extended water structure in CaCl_2_ corresponds to less decrease of *H* (Fig. 3e) and less increase of *-TS* (Fig. 3h), and overall increase in *G* (Fig. 3b) for *r* > 1.7 nm compared to in DI water or MgCl_2_ solution, which indicates higher repulsion. The detailed breakdown for the pair interactions among carbohydrate/water/ions can be found in (Supplementary Discussion). It is interesting to point out that we observed no appreciable increase in electrostatic repulsion from the bound Ca^2+^ ions on surfaces (Fig. S1b,e).

Inspecting the synergistic effects of temperature and specific ions, the complexing of Ca^2+^ ions to carbohydrate surfaces results in less decrease of dehydration energy for the first hydration layer (regime III) at 90 °C where the equilibrium surface distances remain the same as at room temperature (Fig. 3b inset). In contrast, in deionized water or MgCl_2_ solution at 90 °C, the lower dehydration energy for the first hydration layer shortens the equilibrium surface distances (Fig. 3a,c insets). This result suggested that carbohydrates have better hydration and thermal stability in CaCl_2_ solution compared to in MgCl_2_ solution or in deionized water at 90 °C.

## Discussion

**Figure 5:**
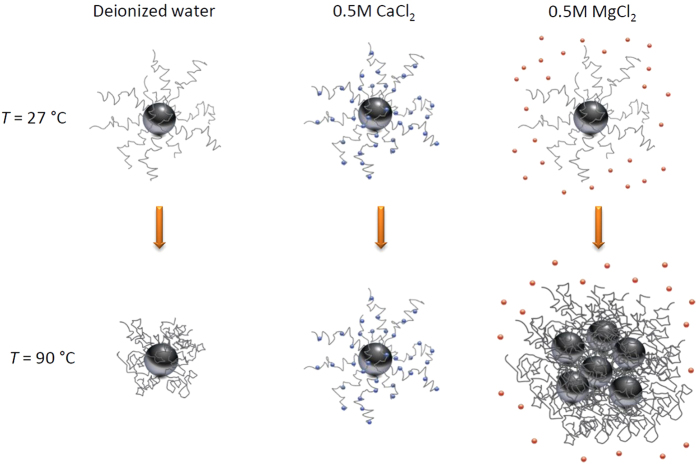
Schematic of proposed mechanism for dextran-coated nanoparticles heated from room temperature to 90 °C in deionized water or electrolyte solutions. Nanoparticle cores are shown as dark gray spheres; dextran coatings are shown as light gray strands; Ca^2+^ ions are shown as blue dots; Mg^2+^ ions are shown as orange dots.

The unified picture between macroscopic experiments and microscopic simulations on the hydration of carbohydrates mediated by temperature and specific ions is summarized in Fig. 5. One important distinction to keep in mind is that the experiments on dextran-coated nanoparticles reflect the collective behavior of carbohydrate polymer coating, while simulations only consider interactions between surfaces with carbohydrate monomers. Thus, the calculated hydration repulsion in simulations is best interpreted as the repulsion between polymer strands on nanoparticles, which indicates the propensity for polymer collapse. At room temperature, the nanoparticles retained their respective sizes in all solutions within the 40 day DLS observation window. The absence of polymer coating collapse and nanoparticle aggregation, despite the slightly negative Δ*G* at the distance of the second hydration layer (Fig. 3a–c for the *T* = 27 °C curves), can be rationalized by the polymeric nature of the nanoparticle coatings. The loss of polymer configurational entropy upon nanoparticle-nanoparticle approach stabilizes the particles against aggregation as long as the first hydration layer on the macromolecules is intact and the polymers remain swelled.

At *T* = 90 °C, the DLS data show three distinct behaviors: the nanoparticles kept the same size as in room temperature in CaCl_2_ solution, decreased in size in deionized water (suggesting polymer collapse), and increased in size in MgCl_2_ solution (suggesting nanoparticle-nanoparticle aggregation) (Fig. 5). The simulations nicely captured the invariance of the local minima of the free energy curves at both *T* = 27 and 90 °C in CaCl_2_ solution (Fig. 3b) that suggest invariant polysaccharide swelling behavior and subsequent invariant nanoparticle sizes. However, despite correct indication of the weaker hydration compared to in CaCl_2_ solution, the simulations showed indistinguishable free energy curves in deionized water and in MgCl_2_ solution at *T* = 90 °C (Fig. 3a,c). The further aggregation of nanoparticles in MgCl_2_ solution at 90 °C compared to the lack of aggregation in deionized water may be due to the fact that the excess non-complexing Mg^2+^ ions can interfere with the hydrophobic hydration of the dextran by increasing the surface tension of the cavity surrounding the backbone and the aliphatic patch of carbohydrate^19^, an effect yet differentiated in our simulations. Nevertheless, the consistent particle sizes in CaCl_2_ solution at different temperatures in experiments confirmed our simulation results that the carbohydrates have optimal hydration repulsion in the presence of surface-complexing Ca^2+^ ions.

Carbohydrates represent an interesting class of molecules with very strong hydration. In fact, there are no LCST transitions for carbohydrate polymers below their boiling point in water, while one of the more broadly used water soluble polymers polyethylene glycol (PEG) has an LCST around 90 to 100 °C in pure water^36^. The optimal thermal stability (strong hydration at high temperatures) of carbohydrates has many technological and biological implications. Industrially, polysaccharides have been used as high temperature viscosifiers. Recent demonstrations show that the nonionic polysaccharide schizophyllan can maintain its high viscosity (i.e. remain hydrated without aggregation or precipitation) in solution at temperatures as high as ~135 to 140 °C, outperforming many other materials for similar applications^37^,^38^. Schizophyllan has also been shown to have high salt tolerance. Interestingly, for experiments investigating the salt tolerance of schizophyllan, a synthetic brine composition with high Ca^2+^ concentration has been used^37^,^38^. Based on the results from our present study, we suspect those Ca^2+^ ions may in fact provide extra hydration mediating the solubility of the polysaccharides in their experiments. Biologically, cell membranes of many extremophilic (organisms that thrive in physically or geochemically extreme conditions, such as those that thrive in high temperature (>80 °C), known as “hyperthermophiles” or high salinity “halophiles”) archaea have been observed to contain high concentrations of glycolipids^39^,^40^. Nevertheless, the direct relations between the glycolipids and the thermal or salt tolerance of extremophiles have yet to be discovered. Another intriguing coincidence is that many physiological functions of the membranes of extremophiles seem to be related to their bound calcium ions, even though the binding sites and the roles of the ions themselves are unclear^41^,^42^. We hypothesize that the increased hydration from calcium binding to glycolipids, thus stabilizing the membrane structure, may provide an alternative view for explaining extremophile physiology. By studying the synergistic temperature and ion-specific effect on carbohydrate hydration, we hope to shed light on the biochemical functions of the glycolipids of extremophiles as well as on the high temperature and salt tolerance of polysaccharides such as schizophyllan. We believe the fundamental understanding of these systems may have broader implications and provide us with a unique and improved approach for the rational design of materials with different applications, especially those employed under extreme conditions.

In summary, in this work we present experimental evidence that polysaccharide (dextran) coated nanoparticles show different long-term colloidal stability and swelling mechanisms at elevated temperature in deionized water, CaCl_2_, and MgCl_2_ solutions, where Ca^2+^ ions promote better hydration for polysaccharide coatings while Mg^2+^ ions induce nanoparticle-nanoparticle aggregation. Microscopic simulations further reveal that the improved carbohydrate hydration in CaCl_2_ solution is due to specific binding of Ca^2+^ ions to carbohydrate surfaces. Surface bound Ca^2+^ ions increase hydration repulsion by increasing the dehydration energy for the first water shell as well as by inducing extended water structures (for at least three water shells) on carbohydrates. The specific motivation of this work stems from recent efforts to deploy functional nanoparticles into subsurface oil reservoirs for hydrocarbon exploration and production applications^43^,^44^,^45^. Due to the harsh subterranean conditions of oil reservoirs, namely high temperature (>90 °C) and salt concentration (>120 000 ppm of total dissolved solids), it has been notoriously difficult to stabilize colloids in these systems. In this study, we show direct binding of Ca^2+^ ions (which are components of connate water) to the carbohydrate periphery, resulting in large hydration repulsion. We believe this work demonstrates the significance of considering specific ion effects rather than overall ionic strength as a key parameter in colloidal stability. Leveraging the binding of highly hydrated (kosmotropic) ions to macromolecular surfaces (such as Ca^2+^ binding to carbohydrates) may represent a new archetype in achieving ultimate hydration of materials with pivotal applications and implications in a wide range of fields.

## Methods

### Reagents and Materials

Iron(II) chloride tetrahydrate, Iron(III) chloride hexahydrate, sodium borohydride, sodium hydroxide, 30% ammonium hydroxide, Tris buffer (2 M), magnesium chloride hexahydrate, calcium chloride hexahydrate and dextran-low fraction for biochemistry (M_w_ ~ 90 kDa) were obtained from Fisher Scientific (Fair Lawn, NJ) and used as received. Pentaerythritol glycidyl ether was obtained from Frontier Scientific, Inc. (Logan, UT) and used as received. Water was double-deionized using a Millipore Milli-Q system to produce 18 MΩ deionized water. Sealable 5 mL microwave vials (CG-4920- 01) were obtained from Chemglass, Inc. (Vineland, NJ) and used as received. Stock electrolyte solutions were prepared by dissolving the appropriate amount of electrolyte into DI H_2_O followed by filtration through a 0.2 μm nylon filter to remove any impurities or dust particles. Tangential flow filtration was performed using a KrosFlo Research II*i* TFF system from Spectrum Labs, Inc. (Rancho Dominguez, CA).

### Synthesis of Dextran-Coated Superparamagnetic Iron Oxide Nanoparticles (Dex-SPIONs)

Dex-SPIONs were prepared using previously reported procedure^46^ with slight modification. In this approach, 1.35 g (0.005 moles) of FeCl_3_·6H_2_O was dissolved in 50 mL of deionized water. To this solution was added 3.0 g of 90 kDa M_w_ dextran followed by cooling of the reaction vessel to 5 °C through the use of an ice water bath and subsequent deoxygenation through the use of an N_2_ purge. This deoxygenation/cooling cycle was applied for 30 minutes while vigorously stirring the reaction vessel with a magnetic stir bar. After 30 minutes, 0.54 g (0.0027 moles) of FeCl_2_·4H_2_O dissolved in 5 mL of deionized water was added to the vessel. The mixture was allowed to stir under an N_2_ atmosphere for an additional 10 minutes. Next, 3 mL of 30% ammonium hydroxide solution was added dropwise to the solution over a period of 15 minutes. During the addition, the reaction color changed from orange to dark brown/black. The reaction was heated to 80 °C for 45 minutes. After heating, the reaction was allowed to cool to room temperature. At this point, the resulting particles are coated non-covalently with a dextran sheath but require crosslinking to ensure that the coating will remain intact during operations. In order to facilitate crosslinking, 2 mL of pentaerythritol glycidyl ether was added to 200 mL of 1 M NaOH (aq) and 400 mg of NaBH_4_ in a round bottom flask. The crude nanoparticle solution was transferred to an addition funnel which was subsequently mounted to the round bottom flask containing the crosslinking formulation. The nanoparticle solution (58 mL) was added dropwise over a period of approximately 1 hour to the vigorously stirring crosslinking solution. The reaction was allowed to proceed at room temperature for 24 hours. Upon completion of the 24 hour reaction period, 20 ml of 2 M 2-amino-2-hydroxymethyl-propane-1,3-diol was added to the crude mixture to quench any unreacted crosslinker present in the medium. This reaction was allowed to proceed for 12 hours. Upon completion, the reaction was purified via tangential flow filtration (100 K MWCO filter) to provide a purified nanoparticle solution with a nominal concentration of 2,250 ppm (280 mL).

### Colloidal Stability Testing Protocol with Dynamic Light Scattering Analysis

Solutions were prepared by adding a 444 μl aliquot of stock Dex-SPION solution to 4.556 mL of the respective 0.5 M electrolyte solutions or DI H_2_O to yield 200 ppm nanoparticle solutions. The solutions were placed inside 5 mL microwave vials which were subsequently crimp- sealed with PTFE lined aluminum septa. Three replicates of each nanoparticle solution were placed in a thermostat regulated oven operating at 90 °C. An additional set of duplicates of each nanoparticle solution were kept at room temperature to serve as control samples.

After the specified time interval, solutions were removed from the oven and allowed to cool to room temperature prior to analysis. Vials were de-crimped and the solutions were transferred to polystyrene cuvettes (BI-SCP, Brookhaven Instruments Co.) for Dynamic light scattering (DLS) analysis. Dynamic light scattering experiments (intensity average) were performed using a Brookhaven NanoBrook system (Brookhaven Instruments Co.) operating at a measurement angle of 90°. The CONTIN algorithm was used for fitting of the autocorrelation functions. Hydrodynamic diameters were calculated using the Stokes-Einstein equation. Each sample was measured in triplicate with an acquisition time of 2 min/sample and a count rate of at least 500 kcps. Differences in salt solution viscosity were not adjusted or accounted for in the measurements, thus the relative change in hydrodynamic diameter is most informative. After measurement, the samples were transferred back to the 5 mL microwave vials, sealed and placed in the oven or benchtop (room temperature samples).

### Molecular Dynamics Simulations

We prepared our model carbohydrate surfaces by attaching carbohydrate monomers (α-D-allopyranose) to zero-charge graphene plates. The O-6 on the carbohydrate monomer was bonded to the carbon atom of the graphene plate, but otherwise, the monomer could freely move. We picked this specific carbohydrate monomer because its structure contains a characteristic *ax-eq-ax* sequence of oxygen atoms on C-1, C-2 and C-3 and has well-established binding constants to specific ions^33^. In the simulations, the carbohydrate monomers and the graphene plates were described based on the CHARMM force field^47^,^48^ with the SPC/E model of water^49^. The ions (Ca^2+^, Mg^2+^, and Cl^−^) were modeled by the recently developed force field of Mamatkulov *et al*.^50^ in which both single-ion and ion-pair properties were considered. The Lorentz-Berthelot combination rule were employed for most of the pairwise LJ potentials from atom-wise ions or CHARMM parameters. However, to reproduce the binding constants between carbohydrate monomers and the different ions, a modified mixing rule was used to reparametrize the interactions between the cations (Ca^2+^ and Mg^2+^) and oxygen atoms on the hydroxyl groups of the carbohydrate monomers^34^. Briefly, the crossed LJ effective radius can be written as σ_Ca(Mg)-O_ = λ_Ca(Mg)-O_(σ_Ca(Mg)_ + σ_O_)/2 where λ_Ca(Mg)-O_ is a fitting parameter, and σ_Ca(Mg)_ and σ_O_ are the LJ radius for individual cations (Ca^2+^ or Mg^2+^) and the carbohydrate oxygens. We used λ_Ca-O_ = 0.92 and λ_Mg-O_ = 1.6 which reproduce the experimental cation-carbohydrate stability constants (*K* = 5.1‒6.5 M^−1^ for Ca^2+^ and *K* < 0.1 M^−1^ for Mg^2+^ complexes)^33^.

A series of extensive molecular dynamics (MD) simulations with different inter-plate distances *r* were carried out under *NPT* condition to obtain hydration pressures and system potentials. For each inter-plate distance, the two carbohydrate surfaces, having a size of 4 × 4 nm^2^ and 16 tethered carbohydrate monomers, were placed around the center of a cubic simulation box at a designed inter-plate distance and subsequently solvated by 6300 water molecules with initial box size 6 × 6 × 6 nm^3^ and periodic boundaries in three dimensions. For the electrolyte solutions, 60 cations (Ca^2+^ or Mg^2+^) and 120 anions (Cl^−^) were randomly placed to substitute the water molecules with the final CaCl_2_ or MgCl_2_ concentration equals to ~0.5 M. The simulation time for each MD run was 100 ns, and the data analysis was performed for the last 60 ns. Statistical uncertainties for pressures were calculated by dividing the data analysis period of the runs into 3 blocks (40 to 60, 60 to 80, and 80 to 100 ns) and obtaining the standard deviation of the block averages. For each complete pressure-distance curve Π(*r*), 211 independent inter-plate distances of 100 ns MD runs were performed for *r* from 2.9 nm to 0.8 nm with 0.01 nm increments, and the total simulation time for one Π(*r*) was 21.1 μs. In this study, the hydration repulsion between carbohydrate surfaces was studied in different solutions and temperatures. Six Π(*r*) curves were calculated under the following conditions: DI water at 27 °C, 0.5 M CaCl_2_ at 27 °C, 0.5 M MgCl_2_ at 27 °C, DI water at 90 °C, 0.5 M CaCl_2_ at 90 °C, and 0.5 M MgCl_2_ at 90 °C; and the total simulation time in this study was 126.6 μs. The hydrostatic pressure was set to 1 bar for all conditions.

All simulations were carried out using *GROMACS* v 4.6.5^51^. Electrostatic interactions were calculated using the particle-mesh Ewald (PME) summation, with a real-space cutoff of 0.9 nm, a grid spacing of 0.12 nm, and fourth-order interpolation. The van der Waals and neighbor-list cutoffs were both set to 0.9 nm. We used velocity rescaling temperature coupling with a time constant of 0.1 ps and Parrinello-Rahman isotropic pressure coupling with a time constant of 2 ps. The simulation time step was set to 2 fs with data collection every 2 ps. Before the production runs, a steepest-descent energy minimization and a 0.1 ns *NVT* simulation were applied to equilibrate the system. All bonds were constrained using the LINCS algorithm^52^ with the exception of water molecules, which were constrained using SETTLE algorithm^53^.

## Additional Information

**How to cite this article**: Chen, H. *et al*. Hydration Repulsion between Carbohydrate Surfaces Mediated by Temperature and Specific Ions. *Sci. Rep.* **6**, 28553; doi: 10.1038/srep28553 (2016).

## Supplementary Material

Supplementary Information
