# Supplementary material for: Hydration Repulsion between Carbohydrate Surfaces Mediated by Temperature and Specific Ions
Source: Sci Rep. 2016 Jun 23;6:28553. doi: 10.1038/srep28553 (PMC4917866; doi:10.1038/srep28553)
Supplement: Supplementary Information [file srep28553-s1.pdf]

Supplementary information for:

Hydration Repulsion between Carbohydrate Surfaces Mediated by Temperature and Specific Ions

Hsieh Chen[1,*,‡], Jason R Cox[1,‡], Hooisweng Ow[1], Rena Shi[1], and Athanassios Z Panagiotopoulos[2]

[1]Aramco Services Company: Aramco Research Center – Boston, Cambridge, MA 02139, USA

[2]Department of Chemical and Biological Engineering, Princeton University, Princeton, NJ 08544, USA

[*]hsieh.chen@aramcoservices.com

[‡]These authors contributed equally to this work

Supplementary Discussion

Pair interaction potentials affected by temperature and specific ions

Figure S1 shows the pair potentials U_{pair} for components in simulations which yields even finer atomistic interaction details. At r = 1.2 nm for the DI water case (Fig. S1a and d), we see $U_{Water-Carb}$ = 37.7, $U_{Water-Water}$ = -5.7 and $U_{Carb-Carb}$ = 19.5 kJ/mol/nm^2 at 27°C; $U_{Water-Carb}$ = 49.9, $U_{Water-Water}$ = -17.1 and $U_{Carb-Carb}$ = 7.1 kJ/mol/nm^2 at 90 °C. The higher $U_{Water-Carb}$ and lower $U_{Water-Water}$ at 90 °C indicate that there are more water-water interactions and less water-carbohydrate interactions, direct evidence that the carbohydrates are less ‘hydrophilic’ and have lower hydration repulsion. As a result, there are more carbohydrate-carbohydrate interactions at high temperature as observed for the lower $U_{Carb-Carb}$ at 90 °C.

To gain a clear picture how the surface binding Ca^{2+} ions effect hydration repulsion, it is instructive to analyze the Ca^{2+}-carbohydrate coordination number (Fig. S1g) and water/carbohydrate/Ca^{2+} ion pair potentials (Fig. S1b and e). With decreasing r the Ca^{2+}-carbohydrate complexing decreases. The removal of Ca^{2+} ions from carbohydrate surfaces to bulk water increases $U_{Ca-Carb}$ and decreases $U_{Ca-Water}$, while, in the meantime, the removal of Ca^{2+} ions from surface corresponds to the addition of waters to the surface which decreases $U_{Water-Carb}$ and increases $U_{Water-Water}$. In contrast, in $MgCl_2$ solution the Mg^{2+}-carbohydrate binding is minimum (Fig. S1h), and the U_{pair} is negligible for all pair interactions with ions (Fig. S1c and f).

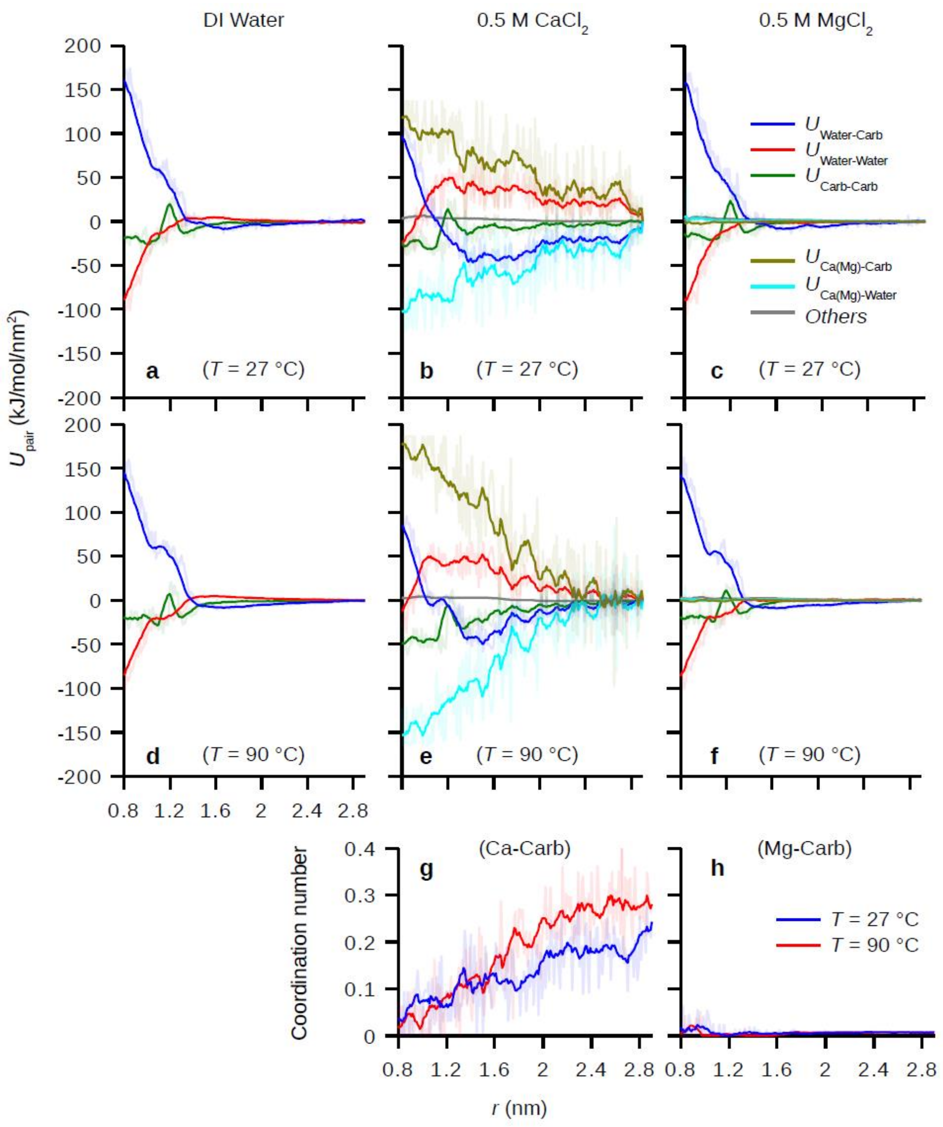

Figure S1. Pair interaction potential between components and Ca^{2+}(Mg^{2+})-carbohydrate coordination numbers. (**a-f**) Pair interaction potentials U_{pair} for different components in simulations at T = 27 ºC (**a-c**) or T = 90 ºC (**d-f**) in DI water (**a,d**), 0.5 M $CaCl_2$ (**b,e**), and 0.5 M $MgCl_2$ (**c,f**). *Others* are the summation of $U_{Carb\text{-}Cl}$, $U_{Ca(Mg)\text{-}Cl}$, $U_{Ca(Mg)\text{-}Ca(Mg)}$, $U_{Cl\text{-}Cl}$, and $U_{Water\text{-}Cl}$ which are all insignificant. (**g,h**) Coordination numbers for Ca^{2+}-carbohydrate complexes (**g**) or Mg^{2+}-carbohydrate complexes (**h**) calculated from integrating the radial distribution function $g(r_{Ca\text{-}Carb})$ or $g(r_{Mg\text{-}Carb})$ from 0 to the first local minimum. Note here the solid curves are the running averages for 10 neighboring original data shown with light colors.
